# Everybody's Page

**Published:** 1888-08-04

**Authors:** 


### Everybody's Page.

The habit of going to bed and getting up at a fixed hour cannot be too highly recommended as a law of health. Regularity in sleeping is as important as regularity in eating.

English people who frequent the Riviera in winter will be glad to hear that the municipal council of Cannes have determined to construct a new and satisfactory system of drainage, which will take away from the town a longstanding reproach.

Dr. T. H. Cullimore recommends lavish indulgence in the switch-back railway and tobogganing as a remedy for biliousness and a substitute for the often unattainable visit to a watering-place. The home-staying schoolboy and the promoters of the various exhibitions should unite in blessing Dr. Cullimore.

Great are the wonders of the telephone! An American physician reports how he was saved a two-mile ride in a driving storm by having the patient, a child, brought to the instrument and held there till it coughed. He diagnosed false croup, prescribed accordingly, and went to bed comfortably. Next morning he found the patient doing very well—under the care of the rival practitioner.

It is said that in 1886 no fewer than 22,134 human beings died from snake-bite in India, and the number of cattle killed by snakes was 2,514; 417,596 snakes were destroyed, and 25,360 rupees were paid by the Goverment as rewards for their destruction. Nevertheless, the snake is not an aggressive animal; he never goes out of his way to bite anyone, but he is prompt to resent the most trifling and unconscious interference with his comfort, and his resentment is fatal.

Dr. Landolt, of Paris, has proposed a method for treating strabismus—which the unlearned call squint—without operation, by resting the eyes, using convex spectacles for convergent strabismus, and looking through the stereoscope for the divergent form, where the eye gazes round the corner, if one may so express it. All these exercises tend to compel the eye to look in the normal direction, and it is said that if begun in time complete success follows these attempts to train up the eye in the way it should go.

It is said that hysteria is becoming more common among the negroes of the United States. Their low form of intelligence and their emotional nature are both favourable to its development, and now that negro women are going in for the tight dresses and the severe course of education of the American woman—for both of which things generations of gradual training will be necessary—as well as the hard physical work that is usually incumbent on them, the results on their health threaten to be serious.

The Rubbi family, in Venice, have been famous glass blowers for nearly four centuries. Their speciality is the manufacture of glass eyes, which they make in all varieties of quality. Common glass eyes, such as are made for hospitals, are easily made, and cost about eight shillings each But fashionable people are not satisfied with these, and some have half a dozen eyes manufactured for them before they are satisfied. Then they require at least two sets of eyes, one for evening wear, with larger pupils than the day ones, because the pupil of the eye is larger by night. Think of the horror of a lady whom some accident has forced to wear a glass eye, on finding after she had entered a ball room, that she had put in the wrong eye, and was going about with pupils of different sizes. The effect would be as bad as a squint, or even a chronic wink.

Paris is suffering again, as it does in most summers, from an insufficient water supply.

Old people should remember that dyspepsia is their most redoubtable foe. Every transgression against the laws of digestion is a nail in their coffin.

Dr. B. O. Kinnear, of Boston, Mass., is of opinion that hay fever is a nervous disease, and has successfully treated several cases on that hypothesis.

Plantations of roses on a large scale are to be established in the Province of Kutais in the Caucasus, so that there may be an extensive native manufacture of otto of roses. At present this is largely imported from Turkey and Bulgaria into Russia, and it is the object of the Minister of Domains, in promoting this enterprise, to oust the foreigner and substitute native industry.

Attempts to find the microbe which will kill the Australian rabbits are being vigorously prosecuted. Pasteur's pupils, Drs. Hind, Lori, and Garmont, have arrived at Sydney and begun cultivating their chicken-cholera germs. Their intention is to first—*à la* Meg Dods—catch their rabbit, inoculate him with the disease whose bacillus most doth please them by its deadly action, and then set him free to infect his friends and relatives. Doubtless extermination is necessary, but this scientific slaughter seems like taking a mean advantage of poor Bunny.

In our issue of last week a story was told of a patient cured by a thermometer. The *Medical Record* of July 14th narrates a case in which the patient's faith in the thermometer nearly killed him. He was a German, and could understand but little English, so when the thermometer was placed in his mouth he thought he was expected to swallow it, which he did, with one mighty gulp. Fortunately the doctor in attendance, horrified at what the patient had done, was successful in his immediate and energetic efforts to regain possession of the missing thermometer, and the man was none the worse for the incident. Since that day, however, the doctor has always tied a thread to the thermometer when taking the temperature of such exceptionally willing and intelligent cases.

The effects of gymnastics on insanity, arising from organic disease of the brain, have been, as yet, but little studied. It is unlikely that any relief in such disease would be afforded by working upon the cerebral nerves. But where instanity is known to have arisen from pressure, from imperfect circulation, or from derangement of some other parts, which may be reached and handled by gymnastics, a cure is often effected, the madness disappearing with the removal of the physical derangement; the quieting effect of the movements on the nerves assisting the cure. There is evidently a rich field opened for investigation in both these directions, with the prospect of great results, if taken up as a special branch of the system. This is the opinion of Miss Ellen F. White, an American lady, who has studied the subject of insanity and its cure. Her suggestion that certain brain-nerves may be brought into a healthy condition by operating upon the spinal system, through properly directed movements, seems highly probable. The beneficial effects of massage, which is a form of passive treatment, are admitted; but other forms of passive treatment might be even more highly productive of good results in the relief of pain, the toning-up of the muscular system, the increase of appetite, and the production of a pleasurable fatigue calculated to induce natural sleep without resort to the use of hypnotics.

